# The effect of adopting pediatric protocols in adolescents and young adults with acute lymphoblastic leukemia in pediatric vs adult centers: An IMPACT Cohort study

**DOI:** 10.1002/cam4.2096

**Published:** 2019-03-26

**Authors:** Sumit Gupta, Jason D. Pole, Nancy N. Baxter, Rinku Sutradhar, Cindy Lau, Chenthila Nagamuthu, Paul C. Nathan

**Affiliations:** ^1^ Division of Hematology/Oncology The Hospital for Sick Children Toronto Ontario Canada; ^2^ Faculty of Medicine University of Toronto Toronto Ontario Canada; ^3^ Cancer Research Program Institute for Clinical Evaluative Sciences Toronto Ontario Canada; ^4^ Institute for Health Policy Evaluation and Management University of Toronto Toronto Ontario Canada; ^5^ Pediatric Oncology Group of Ontario 480 University Ave Toronto Ontario Canada; ^6^ Dalla Lana School of Public Health University of Toronto Toronto Ontario Canada; ^7^ Department of General Surgery Li Ka Shing Knowledge Institute St. Michael's Hospital Toronto Ontario Canada

**Keywords:** acute lymphobastic leukemia, adolescents and young adults, locus of care, population‐based, survival

## Abstract

**Background:**

Retrospective studies have shown adolescents and young adults (AYA) with acute lymphoblastic leukemia (ALL) have superior survival when treated in pediatric versus adult centers (locus of care; LOC). Several adult centers recently adopted pediatric protocols. Whether this has narrowed LOC disparities in real–world settings is unknown.

**Methods:**

The IMPACT Cohort is an Ontario population–based cohort that captured demographic, disease and treatment (treatment protocol, chemotherapy doses) data for all 15‐21 year olds diagnosed with ALL 1992‐2011. Cancer outcomes were determined by chart abstraction and linkage to provincial healthcare databases. Treatment protocols were classified as pediatric‐ or adult‐based. We examined predictors of outcome, including LOC, protocol, disease biology, and time period.

**Results:**

Of 271 patients, 152 (56%) received therapy at adult centers. 5‐year event‐free survival (EFS ± SE) among AYA at pediatric vs adult centers was 72% ± 4% vs 56% ± 4% (*P* = 0.03); 5‐year overall survival (OS) was 82% ± 4% vs 64% ± 4% (*P* < 0.001). After adjustment, OS remained inferior at adult centers (hazard ratio 2.5; 95% confidence interval 1.1‐6.1; *P* = 0.04). In the most recent period (2006‐2011), 39/59 (66%) AYA treated at adult centers received pediatric protocols. These AYA had outcomes superior to the 20 AYA treated on adult protocols, but inferior to the 44 AYA treated at pediatric centers (EFS 72% ± 5% vs 60% ± 9% vs 81% ± 6%; *P* = 0.02; OS 77% ± 7% vs 65% ± 11% vs 91% ± 4%; *P* = 0.004). Induction deaths and treatment–related mortality did not vary by LOC.

**Conclusions:**

Survival disparities between AYA with ALL treated in pediatric vs adult centers have persisted over time, partially attributable to incomplete adoption of pediatric protocols by adult centers. Although pediatric protocol use has improved survival, residual disparities remain, perhaps due to other differences in care between adult and pediatric centers.

## 1 INTRODUCTION

Adolescents and young adults (AYA) with cancer are a vulnerable group whose epidemiology differs from that of older adults and children.^1^ Outcomes among AYA with acute lymphoblastic leukemia (ALL) are particularly poor, especially when compared to the excellent survival experienced by younger children with ALL.^2^ Disease and patient biology, patient behavior, and healthcare system factors have all been suggested as possible explanations for this disparity.^3^

Retrospective studies demonstrated superior AYA ALL outcomes using intensive pediatric treatment protocols as compared to less intensive adult protocols, with differences in event‐free survival (EFS) of 20% or greater.^4^ However, it is unclear to what degree the differences in outcomes were due to the protocols themselves versus other differences between pediatric and adult centers, either in the care provided or population treated. Subsequent prospective studies examined the feasibility of providing pediatric–based treatment protocols to AYA regardless of where they receive their cancer care. These studies confirmed the superiority of pediatric protocols as compared to historical controls, leading to calls for them to become standard for AYA.^5^, ^6^, ^7^ Consequently, several adult institutions have switched to using pediatric–based protocols for this population.

It is unclear whether the adoption of pediatric–based protocols in adult centers has abolished disparities in AYA ALL outcomes between pediatric and adult centers and improved outcomes at a population‐level for this group of patients. Using a population–based cohort of AYA in Ontario, Canada, we examined whether adult–based protocols had been widely adopted, and studied AYA ALL outcomes by locus of care (LOC ‐ pediatric vs adult center) and type of protocol (pediatric‐based vs adult‐based). We also aimed to determine whether any persistent disparities were due to factors previously suggested in the literature, including incomplete adoption of pediatric–based protocols.

## 2 METHODS

### 2.1 Study setting

Canadian healthcare is delivered through provincial universal insurance systems in which most physicians operate on a fee‐for‐service basis. Pediatric oncology care in Ontario is delivered through five tertiary centers. Adolescents aged 15‐18 receive care at either pediatric or adult centers, while older AYA nearly always receive care in adult centers. Though individual AYA initiatives exist, no formal network of AYA cancer units exists.

### 2.2 IMPACT cohort

The Initiative to Maximize Progress in Adolescent and Young Adult Cancer (IMPACT) study collected detailed patient, disease, treatment, and outcome data on all AYA aged 15‐21 years diagnosed in Ontario between 1992 and 2011 with one of six malignancy types: acute leukemia, Hodgkin lymphoma, nonHodgkin lymphoma, soft tissue sarcoma, bone sarcoma, and testicular cancer. Details on the IMPACT Cohort methodology have been published previously.^8^ In brief, AYA treated in pediatric centers were identified through an existent pediatric cancer registry (POGONIS)^9^ while AYA treated in an adult center were identified through the Ontario Cancer Registry (OCR) and their clinical data obtained through chart abstraction. Robust protocols for real‐time data review by clinicians ensured quality abstraction. Abstracted variables included malignancy–level data (stage, histology) and cancer events (eg relapse, progression, second malignancies). Pathology and cytogenetic reports were scanned to facilitate centralized verification of findings. Treatment variables included cancer surgeries, radiation (dose/field), chemotherapeutic, biologic and hormonal agents, and stem cell transplantation. Total dose (per m^2^) was calculated for chemotherapies most associated with late effects (eg anthracyclines, alkylating agents). Clinical trial enrollment and treatment protocols were also abstracted. Demographic data were obtained from the Registered Persons Database (RPDB), a provincial vital statistics registry. Death (RPDB) and second malignancies (OCR) were confirmed by chart abstraction. Records prior to death were reviewed to attribute cause of death.

### 2.3 Additional data sources

Patients were linked deterministically to population–based health services databases housed at the Institute for Clinical Evaluative Sciences (ICES) using unique encoded identifiers based on encrypted health card numbers. These health services databases allowed the identification of hospitalizations, emergency room visits, and physician encounters (Table S1). In addition, Cancer Care Ontario maintains the Cancer Activity Level Reporting (ALR) Database, which includes data elements (eg treatment protocol name) pertaining to radiation and systemic therapies delivered through Regional Cancer Centers and many, though not all, of the facilities that administer chemotherapy to patients.

### 2.4 Outcomes

The primary outcomes were event‐free and overall survival (EFS, OS), both measured from the time of initial diagnosis. Events included relapse, progressive disease, death, and subsequent malignancy. Each individual was followed until the occurrence of the event or until censoring at the date of study termination, whichever occurred first. Cancer events that occurred after AYA were discharged from their initial treatment centers may not have been captured by either POGONIS or IMPACT chart abstraction. This was of particular concern in pediatric center AYA, who would have been transitioned to adult centers shortly after completing treatment. Previously validated algorithms using health services data were used to identify additional cancer events.^10^ Investigators reviewed patterns of health care use around each algorithm–identified event to ensure accuracy. Induction death was defined as any death within 28 days of diagnosis, and thus included deaths prior to treatment initiation. Treatment–related mortality (TRM) was defined as any death occurring after diagnosis in the absence of another cancer event (relapse, progressive disease, subsequent malignancy).

### 2.5 Variables

LOC was categorized as pediatric vs adult center, based on the institution that delivered the first 3 months of chemotherapy. For treatment protocol, AYA treated at pediatric centers were categorized as having been treated with pediatric–based protocols, since all five pediatric centers use such regimens. AYA treated at adult centers were categorized as having received pediatric–based vs adult–based protocols using three data sources. Where a specific protocol name was recorded either in the IMPACT database (ie through chart abstraction) or in the ALR, this protocol was categorized by the investigator. Where either no treatment protocol or only a generic treatment protocol was listed, patterns of chemotherapy were examined. During the study time period, several adult institutions in Ontario participated in the Dana‐Farber Cancer Institute (DFCI) ALL Consortium. DFCI treatment involves a prolonged phase of weekly asparaginase administration.^5^ Patients with three or more records for E. Coli asparaginase in any 21 day period within the first 16 weeks post diagnosis but prior to any cancer event were categorized as pediatric–based treatment. All remaining AYA were categorized as receiving adult–based treatment. The proportions of patients receiving pediatric–based treatment at each of the largest adult centers was calculated and then verified with local clinicians to ensure consistency with practice.

Patient–level predictors included age at diagnosis and sex. Neighborhood income quintile was determined using data from the Canadian census closest to date of diagnosis. Urban vs rural status was determined using postal code at diagnosis and the Rurality Index for Ontario 2004. Time period of diagnosis was defined as early (1992‐1998), middle (1999‐2005), or late (2006‐2011). Disease–level variables included white blood cell (WBC) count at presentation, immunophenotype (B vs T), and extramedullary involvement [central nervous system (CNS), testicular]. When available, cytogenetics were categorized as favorable (TEL‐AML, hyperdiploid, or triple trisomy), unfavorable (hypodiploid, Philadelphia chromosome positive, MLL rearrangements), or neutral (all others).

### 2.6 Analyses

The distributions of characteristics among AYA treated at pediatric vs adult centers were compared descriptively, using chi squared tests or Fisher's exact tests for categorical variables as appropriate, and t‐tests for continuous variables. EFS and OS were computed using the Kaplan‐Meier approach, and were compared between care loci and between protocol types with the log rank test. Predictors of EFS and OS were determined using univariate and multivariable Cox Proportional Hazards regression models; predictors significant at the 0.1 level in univariate analyses were included in the multivariable model. Interactions between LOC and time period were examined. Competing risks analyses were used to examine the risk of TRM over time, accounting for other events (relapse, progressive disease, second cancer) as competing events. The cumulative incidence function approach was used to determine the risk of TRM; these risks were compared by loci of care using Gray's test. Other outcomes including induction deaths were assessed in a descriptive manner. Significance was defined as *P* < 0.05. Analyses were performed using SAS, version 9.4 (SAS Institute, Cary, NC). Ethics approval was obtained at multiple institutions, including The Hospital for Sick Children, St. Michael's Hospital, and Sunnybrook Health Sciences Centre. Informed consent was not required.

## 3 RESULTS

Over the study period, 275 AYA were diagnosed with ALL, 152 (55.3%) of whom were treated in adult centers. Demographic and disease characteristics, stratified by LOC, are shown in Table 1. Adult center AYA were treated at 17 institutions. The five largest adult centers accounted for 124/152 (81.6%) of such patients overall, and 46/59 (78.0%) of such patients in the late time period (2006‐2011).

**Table 1:** Demographic and disease characteristics of study cohort (N = 275), stratified by locus of care

	Missing data (N, %)	Pediatric center (N = 123)	Adult center (N = 152)	*P*
Age (years, mean, standard deviation)	0 (0)	15.9 ± 0.9	18.7 ± 1.4	**<0.001**
Sex (N, %)	0 (0)			0.13
Male		81 (65.9)	113 (74.3)	
Female		42 (34.1)	39 (25.7)	
Time period (N, %)	0 (0)			0.06
Early (1992‐1998)		31 (25.2)	53 (34.9)	
Middle (1999‐2005)		48 (39.0)	40 (26.3)	
Late (2006‐2011)		44 (35.8)	59 (38.8)	
Neighborhood income quintile (N, %)	6 (2.2)			
Q1 (lowest)		20 (16.3)	27 (17.8)	0.40
Q2		22 (17.9)	22 (14.5)	
Q3		26 (21.1)	36 (23.7)	
Q4		28 (22.8)	32 (21.1)	
Q5 (highest)		22 (17.9)	34 (22.4)	
Rurality (N, %)	≤5^a^			0.73
Urban		103 (83.7)	130 (85.5)	
Rural		18 (14.6)	21 (13.8)	
WBC at presentation (N, %)	30 (10.9)			0.49
<50 × 10^9^/L		79 (79.0)	109 (75.2)	
≥50 × 10^9^/L		21 (21.0)	36 (24.8)	
Lineage (N, %)	61 (22.2)			0.92
B		58 (75.3)	104 (75.9)	
T		19 (24.7)	33 (24.1)	
CNS involvement	0 (0)			0.66
Negative		115 (93.5)	144 (94.7)	
Positive		8 (6.5)	8 (5.3)	
Testicular involvement	0 (0)			n/a
No		123 (100.0)	152 (100.0)	
Yes		0 (0.0)	0 (0.0)	
Cytogenetics	145 (52.7)			**<0.001**
Favorable		14 (37.8)	5‐10^a^	
Neutral		11 (29.7)	83 (89.2%)	
Unfavorable		12 (32.4)	≤5^a^	
Treatment protocol type (N, %)	0 (0)			
Pediatric‐based		123 (100)	46 (30.3)	**<0.001**
Adult‐based		0 (0)	106 (69.7)	
Registered on clinical trial	0 (0)			
Yes		86 (69.9)	7 (4.6)	**<0.001**
No		37 (30.1)	145 (95.4)	
Stem cell transplant in first remission	0 (0)			
Yes		18 (14.6)	24 (15.8)	0.79
No		105 (85.4)	128 (84.2)	

CNS, central nervous system; IQR, interquartile range; N, number; WBC, white blood cell.

a Privacy regulations prevent the disclosure of small cell sizes ≤ 5.

Bolded values indicate statistically significant values at *P* < 0.05.

AYA treated at a pediatric center were younger than those treated at adult centers (mean 16 ± 1 year vs 19 ± 1 year; *P* < 0.001), but did not differ by other demographic characteristics. Markers of disease biology also did not differ significantly by LOC with the exception of AYA treated at adult centers being more likely to have neutral cytogenetics. AYA at pediatric centers were far more likely to be registered on clinical trials [86/123 (69.9%) vs 7/152 (4.6%); *P* < 0.001] but were no more likely to undergo stem cell transplant in first remission [18/123 (14.6%) vs 24/152 (15.8%); *P* = 0.79].

**Figure 1:**
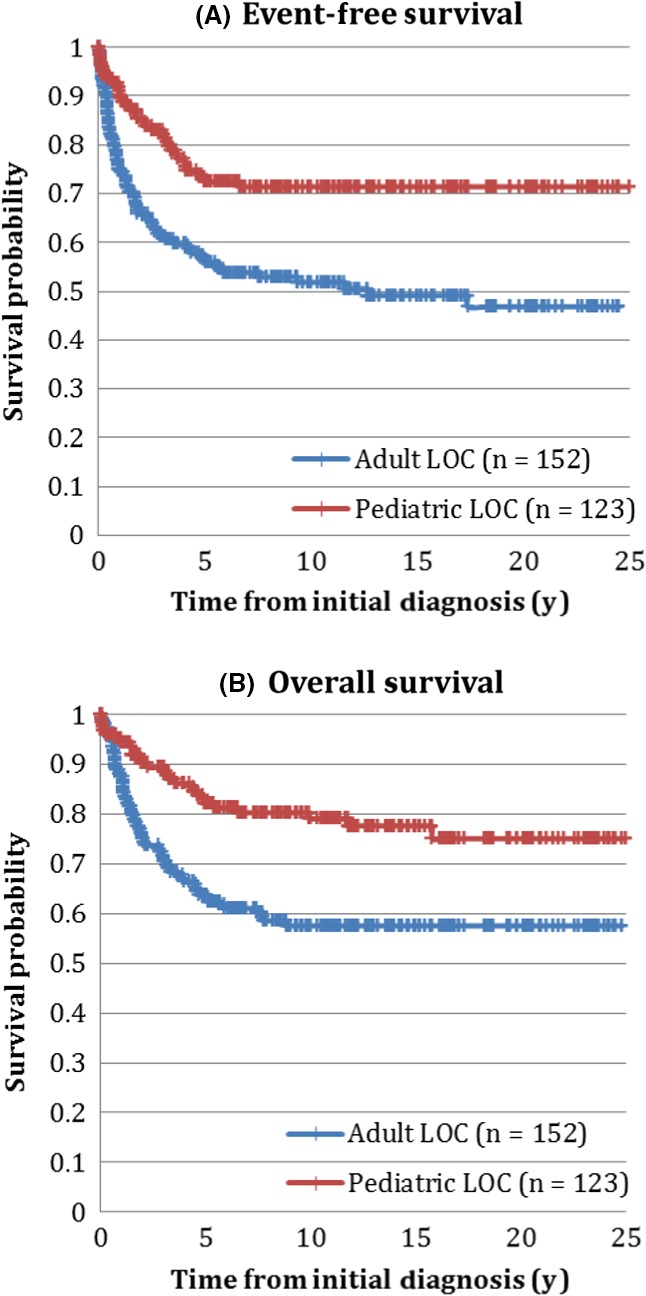
(A) Event‐free and (B) overall survival of adolescents and young adults with acute lymphoblastic leukemia, by locus of care

The 5 year EFS for AYA treated in pediatric centers was 72.4% ± 4.0% vs 56.6% ± 4.0% in AYA treated in adult centers (*P* = 0.03). The respective 5‐year OS was 82.1% ± 3.5% vs 63.8% ± 3.9% (*P* < 0.001) (Figure 1). LOC–based disparities persisted over time (Table 2). For example, the 5‐year OS for patients treated in the latest time period was 90.9% ± 4.3% for those AYA treated in pediatric centers vs 72.9% ± 5.8% for those treated in adult centers (*P* = 0.02).

**Table 2:** 5‐year event‐free and overall survival in adolescents and young adults with acute lymphoblastic leukemia treated at pediatric versus adult centers

	5‐year event‐free survival ± SE			5‐year overall survival ± SE		
	Pediatric center	Adult center	*P* value	Pediatric center	Adult center	*P* value
Overall (N = 275)	72.4 ± 4.0	56.6 ± 4.0	**0.03**	82.1 ± 3.5	63.8 ± 3.9	**0.0006**
Early (1992‐1998) (N = 84)	64.5 ± 8.6	50.9 ± 6.9	0.16	74.2 ± 7.9	58.5 ± 6.8	0.13
Middle (1999‐2005) (N = 88)	68.8 ± 6.7	47.5 ± 7.9	**0.04**	79.2 ± 5.9	57.5 ± 7.8	**0.02**
Late (2006‐2011) (N = 103)	81.8 ± 5.8	67.8 ± 6.1	0.08	90.9 ± 4.3	72.9 ± 5.8	**0.02**

Bolded values indicate statistically significant values at *P* < 0.05.

Predictors of EFS and OS are shown in Table 3. When sociodemographic (including age) and disease–related variables were accounted for, a trend towards inferior EFS in adult centers versus pediatric centers was observed [hazard ratio (HR) 1.92, 95% confidence interval (CI) 0.99‐3.75; *P* = 0.06]. However, OS was statistically significantly inferior in adult centers as compared to pediatric centers (HR 2.54, 95CI 1.06‐6.10; *P* = 0.04). LOC–based disparities persisted over time (Table 2); the magnitude of this disparity did not significantly differ between time periods (*P* value of interaction between LOC and time period >0.05).

**Table 3:** Predictors of event‐free and overall survival in adolescents and young adults with acute lymphoblastic leukemia

	Event‐free survival				Overall survival			
	Univariate		Multivariable (n = 245)		Univariate		Multivariable (n = 201)	
	HR (95th CI)	*P*	HR (95th CI)	*P*	HR (95th CI)	*P*	HR (95th CI)	*P*
Locus of care (n = 275)								
Pediatric	—	—	—	—	—	—	—	—
Adult	**2.07 (1.39‐3.10)**	**<0.001**	1.92 (0.99‐3.75)	0.06	**2.21 (1.41‐3.47)**	**<0.001**	**2.54 (1.06‐6.10)**	**0.04**
Age (per year) (n = 275)	**1.16 (1.06‐1.29)**	**0.003**	1.00 (0.85‐1.18)	1.00	**1.14 (1.02‐1.27)**	**0.02**	0.94 (0.77‐1.14)	0.52
Sex (n = 275)								
Male	—	—	—	—	—	—	—	—
Female	**0.68 (0.44‐1.05)**	**0.08**	**0.55 (0.33‐0.91)**	**0.02**	**0.69 (0.34‐0.94)**	**0.03**	**0.34 (0.16‐072)**	**0.005**
Time period (n = 275)								
Early (1992‐1998)	—	—	—	—	—	—	—	—
Middle (1999‐2005)	0.80 (0.51‐1.24)	0.31	0.80 (0.48‐1.31)	0.37	0.82 (0.51‐1.32)	0.42	0.94 (0.77‐1.14)	0.95
Late (2006‐2011)	**0.51 (0.32‐0.83)**	**0.006**	**0.44 (0.26‐0.75)**	**0.003**	**0.49 (0.29‐0.84)**	**0.009**	**0.45 (0.23‐0.88)**	**0.02**
Neighborhood income quintile (n = 269)								
Q1 (lowest)	—	—	—	—	—	—	—	—
Q2	0.69 (0.35‐1.36)	0.28	—	—	0.69 (0.33‐1.42)	0.31	—	—
Q3	1.01 (0.57‐1.78)	0.99	—	—	0.81 (0.43‐1.52)	0.51	—	—
Q4	1.16 (0.66‐2.06)	0.60	—	—	0.99 (0.53‐1.83)	0.97	—	—
Q5 (highest)	0.70 (0.37‐1.32)	0.27	—	—	0.68 (0.35‐1.34)	0.27	—	—
Rurality (n = 272)								
Urban	—	—	—	—	—	—	—	—
Rural	1.26 (0.76‐2.06)	0.37	—	—	0.71 (0.37‐1.38)	0.31	—	—
WBC at presentation (n = 245)								
<50 × 10^9^/L	—	—	—	—	—	—	—	—
≥50 × 10^9^/L	**1.95 (1.27‐3.01)**	**0.002**	**2.07 (1.31‐3.26)**	**0.002**	**1.98 (1.23‐3.21)**	**0.005**	1.75 (0.95‐3.23)	0.07
Lineage (n = 214)								
B	—	—	—	—	—	—	—	—
T	1.40 (0.88‐2.23)	0.16	—	—	**1.67 (0.37‐0.98)**	**0.04**	1.19 (0.65‐2.15)	0.57
CNS involvement (n = 275)								
Negative	—	—	—	—	—	—	—	—
Positive	**2.27 (1.18‐4.36)**	**0.01**	2.08 (0.97‐4.43)	0.06	**1.93 (0.93‐3.99)**	**0.07**	1.82 (0.61‐5.41)	0.28
Cytogenetics (n = 130)								
Favorable	0.52 (0.21‐1.33)	0.17	—	—	0.53 (0.9‐1.50)	0.23	—	—
Neutral	—	—	—	—	—	—	—	—
Unfavorable	1.36 (0.64‐2.93)	0.42	—	—	0.98 (0.38—2.51)	0.96	—	—

CNS, central nervous system; WBC, white blood cell.

Bolded values indicate statistically significant values at *P* < 0.05.

Of the 152 AYA treated at adult centers, 46 (30.3%) were treated using pediatric–based protocols. As expected, <5 AYA were treated with pediatric–based protocols during each of the early and middle time periods, representing <10% of patients. Of 59 patients treated during the late time period (2006‐2011), 39 (66.1%) were treated using pediatric–based protocols. The proportion of late time period AYA treated with pediatric–based protocols varied by center between 0% and100% (numbers not shown due to small cell sizes). Of the five largest centers by volume, the proportion still varied from 0% to 100%.

**Figure 2:**
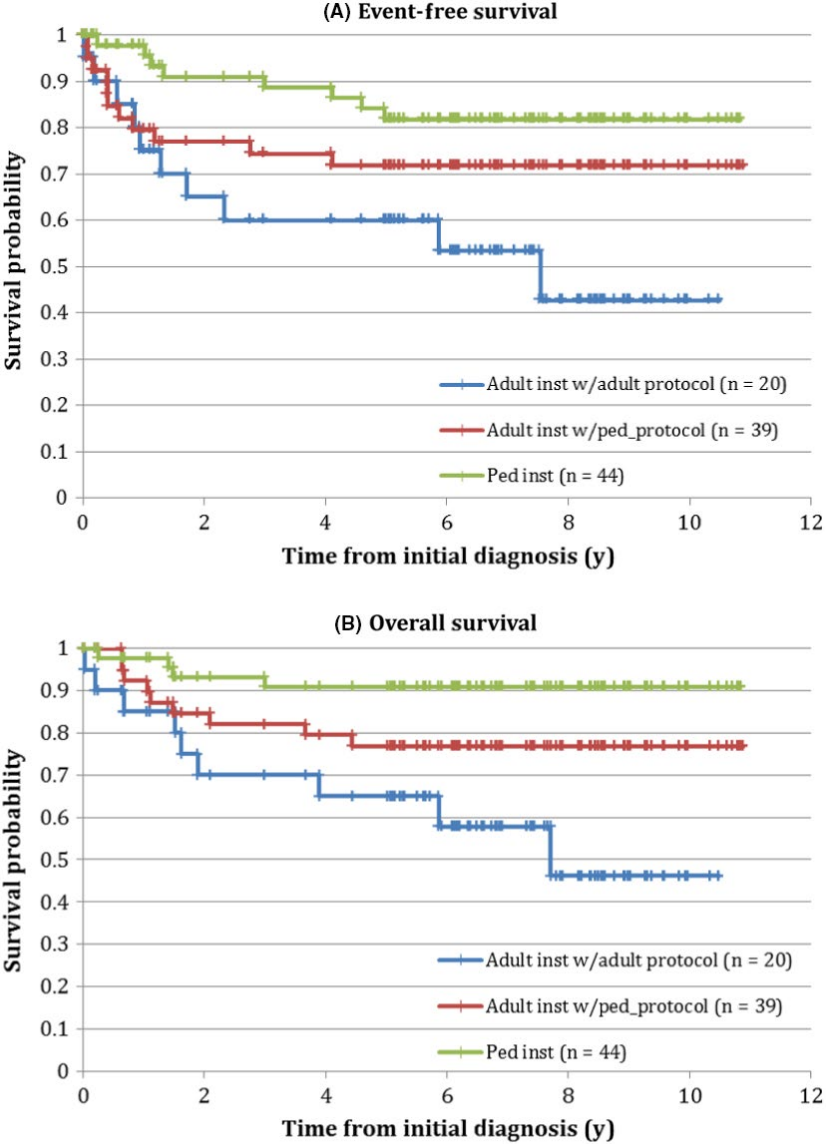
(A) Event‐free and (B) overall survival for adolescents and young adults with acute lymphoblastic leukemia treated during the late time period, by locus of care and protocol type

Given that very few adult center AYA were treated with pediatric–based protocols in the early or middle time period, outcome analyses by protocol type were restricted to the late time period. EFS and OS for pediatric center vs adult center/pediatric–based protocol vs adult center/adult–based protocol are shown in Figure 2. The 5‐year EFS was highest for pediatric center AYA, intermediate for adult center AYA treated with pediatric–based protocols, and lowest for adult center AYA treated with adult–based protocols (80.8% ± 5.8% vs 71.8% ± 7.2% vs 60.0% ± 11.0% respectively; *P* = 0.02). The same disparity was observed in 5‐year OS (90.9 ± 4.3% vs 76.9% ± 6.8% vs 65.0 ± 10.7%; *P* = 0.004).

The proportion of induction deaths did not differ between AYA at pediatric vs adult centers (<2% in both; *P* = 0.44; numbers not shown due to small cell sizes). The 2‐year cumulative incidence of TRM was 5.6% ± 2.1% for pediatric center AYA vs 5.9% ± 1.9% for adult center AYA (*P* = 0.95). Restricted to AYA diagnosed in the late period, the pediatric vs adult 2‐year TRM was 2.3% ± 2.3% vs 3.4% ± 2.4% (*P* = 0.88).

## 4 DISCUSSION

In this population–based study combining clinical and health services data, we found that LOC–based disparities in the survival of AYA with ALL treated at pediatric compared to adult centers have persisted over time despite an overall improvement in outcome. Persistence of these disparities are partially, but not wholly attributable to the incomplete adoption of pediatric–based protocols at adult centers.

Prospective studies have convincingly demonstrated that pediatric–based ALL treatment is associated with improved outcomes in AYA. The Dana‐Farber Cancer Institute (DFCI) Adult ALL Consortium demonstrated that in 18‐50 year old patients with ALL, treatment with a modified DFCI pediatric protocol was associated with a 4‐year disease free survival (DFS) of 69% (CI 56%‐78%).^5^ The US intergroup trial C10403 administered treatment based on the standard arm of the Children's Oncology Group (COG) protocol AALL0232 to AYA ALL patients aged 16‐39 years at diagnosis, and found a 3‐year EFS of 59% (CI 54%‐65%) and a 3‐year OS of 73% (CI 68%‐78%).^7^ In Scandinavia, patients aged 18‐45 years experienced a 5‐year EFS of 74% (CI 70%‐78%) when treated with pediatric Nordic Society of Pediatric Hematology Oncology (NOPHO) protocols.^11^ These represent substantial improvements over historical survival rates of <50% for patients treated with adult–based protocols.^12^ Pediatric protocols generally involve higher cumulative doses of agents such as asparaginase, prednisone, and vincristine. Intensive CNS–directed therapy also plays an key role in pediatric protocols and may also be important in AYA; a recent Japanese trial of adult ALL that randomized patients between intermediate and high–dose methotrexate confirmed the superiority of the latter.^13^

The above results have collectively led to calls for pediatric–based protocols to become standard in AYA with ALL.^14^ Whether this has led to population–based improvements in AYA ALL outcomes has been unclear. Muffly et al showed that in California, the percentage of AYA with ALL treated with pediatric–based protocols at adult institutions had fallen over time, dropping from 31% in 2008‐2012, when C10403 was open to accrual, to 21% in 2013‐14.^15^, ^16^ Institutions that treated ≥2 AYA ALL patients per year were more likely to use pediatric–based treatment. Adoption of pediatric–based treatment was also not universal in our Ontario cohort, though we showed a rapid increase over the study time period. Of patients diagnosed between 2006 and 2011, 66% were treated with such protocols, compared to <10% in earlier time periods. This proportion has likely risen since the end of the study period as additional adult centers have adopted pediatric protocols.

Our results suggest that AYA treated with pediatric–based protocols at adult centers have intermediate outcomes between AYA treated with adult–based protocols and those treated at a pediatric center. These results echo a recent Californian study that showed that among AYA treated in adult settings, outcomes were similar between those treated with pediatric–based vs adult–based protocols; both groups had inferior outcomes compared to AYA at pediatric centers.^16^ It is possible that our finding of intermediate outcomes may be due to differences in disease biology; unsurprisingly, pediatric center AYA were younger than their adult center counterparts (median age at diagnosis of 16 vs 19 years, respectively). However, we did not find significant differences in traditional markers of aggressive disease biology, including WBC at presentation, immunophenotype, and extramedullary involvement between AYA at pediatric vs adult centers. In addition, our cohort represents a relatively narrow and postpubertal age spectrum (15‐21 years), making substantial differences in disease biology unlikely.

It is likely that other differences in care between adult and pediatric centers contribute to differences in outcomes, even when similar treatment protocols are used. Two main mechanisms are suggested in the literature: adherence to dose intensity and differences in psychosocial care. AYA are at higher risk of toxicity from specific chemotherapies, including vincristine and asparaginase.^17^ Pediatric oncologists may be more comfortable with continuing intensive treatment and maintaining dose intensity in the face of such toxicity.^5^ Dose reductions of these agents may have contributed to outcome differences. In the DFCI Protocol 91‐01 for example, patients who tolerated <25 weeks of asparaginase had a significantly inferior EFS (73% ± 7% vs 90% ± 2%; *P* < 0.1). This protocol formed the basis for the AYA protocol adopted by many Ontario adult institutions. Interestingly, AYA at pediatric centers were far more likely to be registered on clinical trials (69.9% vs 4.6%). Participation in clinical trials may be associated with stricter physician adherence to treatment regimens.^18^

Differences in psychosocial and supportive care may also play a role. AYA have been shown to have lower rates of medication adherence as compared to younger children.^19^ In ALL, initial intensive treatments are followed by a long period of less intense maintenance therapy consisting of oral antimetabolites such as 6‐MP. Rates of 6‐MP nonadherence of >5% have been associated with a threefold increase in the risk of relapse.^20^ Given lower patient volumes in pediatric centers, AYA at risk of nonadherence may be more easily identified and referred to the appropriate psychosocial supports. Future studies investigating adherence patterns and interventions in adult center AYA are warranted. It should be noted that LOC–based differences in 6‐MP prescribing patterns on the part of physicians have also not been studied.

Study strengths include the availability of detailed clinical data on a population–based cohort of AYA. Chart abstraction was carried out with real–time validation by clinical experts. Linkages to health administrative data allowed for the identification of additional cancer events and treatment protocols. As noted, our major study limitations were those inherent to retrospective population–based studies, including fixed sample sizes, differences between comparator groups, and the unavailability of certain data allowing for robust conclusions on mechanisms underlying disparities (eg data on patient and physician compliance with treatment). Mechanisms suggested by our results should therefore be considered hypothesis generating and worthy of further study using prospective methods. Small sample sizes also prevented examining potential center–level variations in outcome. Finally, results may not be generalizable to settings without universal health insurance as uninsured status has been associated with disparities in various AYA cancer outcomes.^2^

In conclusion, we have shown that LOC–based disparities have persisted over time. Universal adoption of pediatric–based treatment protocols in adult centers is warranted, potentially through policies mandating the treatment of AYA ALL at specialized centers. Universal adoption should further narrow but may not completely abolish LOC–based disparities. Further studies of the role of supportive and psychosocial care and of dose reductions of important medications like asparaginase are needed.

## CONFLICT OF INTEREST

None declared.
