# Supplementary material for: The effect of adopting pediatric protocols in adolescents and young adults with acute lymphoblastic leukemia in pediatric vs adult centers: An IMPACT Cohort study
Source: Cancer Med. 2019 Mar 26;8(5):2095–103. doi: 10.1002/cam4.2096 (PMC6536996; doi:10.1002/cam4.2096)
Supplement: Supplementary file 1 [file CAM4-8-2095-s001.docx]

**Supplemental Table 1 – Population-based health services databases used to derive high-intensity end-of-life care measures**

| **Database** | **Data Elements** | **Description** | **Initiation Year** |
| --- | --- | --- | --- |
| ALR | Cancer therapies | Data elements pertaining to systemic cancer therapies, including radiation and chemotherapy | 2007 |
| DAD | Inpatient hospitalizations | One record per hospital admission including chart-abstracted demographic, clinical and outcome data. | 1988 |
| NACRS | ED visits | Demographic, clinical and disposition data. | 2000 |
| OHIP | Physician claims | Claims for services billed by fee-for-service Ontario physicians. Physicians under alternative funding plans are also required to submit shadow claims, ensuring capture of nearly all physician encounters. | 1991 |

ALR – Cancer Activity Level Reporting; DAD – Discharge Abstract Database; ED – emergency department; NACRS – National Ambulatory Care Reporting System; OHIP – Ontario Health Insurance Plan Claims Database
